# Polarized resonant emission of monolayer WS_2_ coupled with plasmonic sawtooth nanoslit array

**DOI:** 10.1038/s41467-020-14597-2

**Published:** 2020-02-05

**Authors:** Chunrui Han, Jianting Ye

**Affiliations:** 1 Institute of Microelectronics, Chinese Academy of Sciences, 100029 Beijing, China; 2 Device Physics of Complex Materials, Zernike Institute for Advanced Materials, University of Groningen, Nijenborgh 4, 9747 AG Groningen, The Netherlands

**Keywords:** Two-dimensional materials, Metamaterials

## Abstract

Transition metal dichalcogenide (TMDC) monolayers have enabled important applications in light emitting devices and integrated nanophotonics because of the direct bandgap, spin-valley locking and highly tunable excitonic properties. Nevertheless, the photoluminescence polarization is almost random at room temperature due to the valley decoherence. Here, we show the room temperature control of the polarization states of the excitonic emission by integrating WS_2_ monolayers with a delicately designed metasurface, i.e. a silver sawtooth nanoslit array. The random polarization is transformed to linear when WS_2_ excitons couple with the anisotropic resonant transmission modes that arise from the surface plasmon resonance in the metallic nanostructure. The coupling is found to enhance the valley coherence that contributes to ~30% of the total linear dichroism. Further modulating the transmission modes by optimizing metasurfaces, the total linear dichroism of the plasmon-exciton hybrid system can approach 80%, which prompts the development of photonic devices based on TMDCs.

## Abstract (web summary)

Here the authors show that WS_2_ coupled with a plasmonic sawtooth nanoslit array is an efficient exciton-plasmon hybrid system which enables polarization modulation of the excitonic emission at the nanoscale up to 80% and observation of valley coherence at room temperature.

## Introduction

Transition metal dichalcogenides (TMDCs) have gathered significant scientific interest because of their spectacular optical^1,2^, electronic^3,4^, mechanic^5,6^, and spintronic^7^ properties, especially when reduced to the two-dimensional limit. Monolayer semiconducting TMDCs possess a direct bandgap that exhibits photoluminescence (PL) hundreds of times stronger than that of an indirect one^8^. With proper treatment, the PL quantum yield can approach the unity to guarantee a high radiative recombination rate even at room temperature^9^. The large exciton binding energy leads to strong and long-lived excitons with high photostability allowing for the efficient coupling with external materials^10^. On the other hand, monolayer flakes are easy to be incorporated in a functional device because of the atomically flat and chemically inert surface compatible with nanolithography and stacking processes. Additionally, the highly efficient growth by chemical vapor deposition (CVD) is promising to meet the demand of mass production^11^. Benefiting from these advantages, TMDCs offer a unique platform for investigation of intriguing light-matter interactions at the nanoscale through, besides the well-known van der Waals heterostructures^12,13^, the integration with artificial materials, such as photonic nanocavities^14,15^, plasmonic nanostructures^16^, and single nanoparticle antennas^17^. This enables the establishment of compact optoelectronic devices, including tunable light emitters^18^, nanolasers^19,20^, electro-optic modulator^21^, optical switches^22^, biosensors/detectors^23,24^, field-effect transistors^25^, quantum devices^26^, etc., which are the key elements of the next-generation integrated photonic circuits. Recently, TMDCs implemented in plasmon-exciton hybrid systems have been intensively explored, such as giant Rabi splitting^27,28^, multifold enhancement in PL^29,30^ and active control of plasmon-exciton coupling^31,32^ in various noble metal-TMDC hybrid nanostructures. However, polarization manipulation of the excitonic emission in such hybrid systems has been elusive.

Polarized systems are of crucial importance for applications in material characterization, medical diagnosis, and super resolution imaging^33–35^. Especially, manipulating the polarization of photons at the nanoscale enables technical advancements in the areas of single molecule detection^36^, ultrasensitive sensing^37,38^, quantum communication^39,40^, etc. For bare TMDCs, the linearly polarized emission can only be generated by a coherent superposition of two circularly polarized photons from *K* and *K'* valleys^41^, and the polarization direction can be controlled by the magnetic field induced valley Zeeman splitting effect^42^. However, the intervalley quantum coherence survives only at a few tens of Kelvins and the resulted linearity is not sufficient—it is even worse in ambient conditions, due to the stronger intervalley scattering of carriers by the Coulomb interaction and phonons. The plasmonic metasurface—the broadband and ultrafast plasmonic cavity—is proposed to suppress the quantum decoherence by enhancing the light-matter interactions, which is essential to preserve the linear polarization of the valley excitons at room temperature^43^. In addition, the anisotropic resonant transmission mode, whose near-field resonance is strongly polarized when coupled to the free space, is able to guide the emission and boost the linearity significantly^44,45^.

To control the polarization of the PL emission at the nanoscale, it is essential to identify desirable optical modes in the plasmonic nanostructure. First of all, the optical mode needs to couple with the excitation field, so that the pump energy can be absorbed efficiently by the TMDC monolayers buried underneath the metallic structure. The optical mode should also coincide with the direct excitonic transition, thus the emission field can be confined in subwavelength volumes and driven by the strong oscillations of surface plasmons to radiate intensively to the far field. At last, the optical mode responds distinctly to the orthogonally polarized light, allowing for the formation of a preferable polarization for the coupled excitons.

Here we show that a delicately designed metallic sawtooth nanoslit array, whose configuration supports plasmonic resonances strongly polarized along the long axis of the array, fulfills all the above requirements. In particular, strong resonant transmission can be excited at different wavelengths and precisely tuned by both the dielectric environment and the geometrical parameter of the metasurface, thus providing the possibility to control both excitation and emission paths of TMDC monolayers separately. A large linearity of the PL emission up to 80% has been achieved in which the contribution from the valley coherence is about 30%. Consequently, both polarization and intensity of the PL emission can be well modulated in a sawtooth nanoslits-TMDC hybrid system, enabling ultrathin and flexible polaritonic devices of multifunctionality.

## Results

### Device configuration

**Fig. 1:**
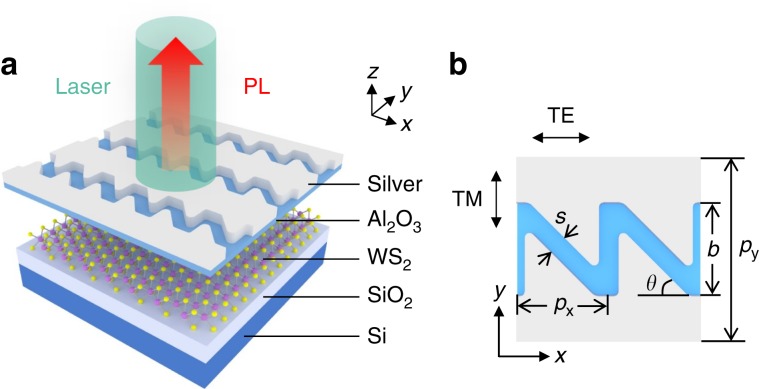
Schematics of the WS_2_-Ag hybrid nanostructure. **a** 3D schematic view of the hybrid plasmonic device consisting of silver sawtooth nanoslit array on top of WS_2_ monolayer separated by a thin layer of Al_2_O_3_. The device is pumped by a 532 nm laser with the incident polarization fixed in the *x* direction. **b** Top view of two unit cells for the sawtooth nanoslit array with geometrical parameters: *p*_*x*_ = 200 nm, *p*_*y*_ = 400 nm, *s* = 40 nm, *b* and *θ* are two variables.

As illustrated in Fig. 1a, the hybrid system consists of a 2*H*-type WS_2_ monolayer on the SiO_2_/Si substrate, a layer of Al_2_O_3_ and the silver sawtooth nanoslit array patterned subsequently on top. The structural parameters of the sawtooth nanoslits are shown in Fig. 1b. Different from most of previous works where the WS_2_ monolayers were transferred on the plasmonic nanostructure, here in our system they were designed to be beneath the metallic layer. This configuration allows adequate interaction between excitons and the plasmonic metasurface to guarantee an efficient plasmonic modulation. In addition, inserting an Al_2_O_3_ layer between the WS_2_ and sawtooth nanoslits isolates the WS_2_ from dopants in the ambient atmosphere and serves as a control parameter for tuning the spectral position of the plasmonic resonance because different thicknesses of Al_2_O_3_ layers would change dielectric environments of the sawtooth nanoslits.

For the metasurface on top, the sawtooth rather than line nanoslits are chosen, because the former supports broadband resonant transmission, which could enable efficient excitation and emission of PL (Supplementary Fig. 1). At the same time, the transmission mode is polarized preferentially along the TE direction (Fig. 1b) which is in parallel with the long axis of the sawtooth nanoslit array, hence is able to drive the PL emission to be polarized along the same direction. Furthermore, the resonant transmission modes are sensitive to the geometrical parameters, e.g. the slanting angle *θ* of the middle slit (Fig. 1b), thereby offering the opportunity for the polarization modulation of the PL emission. To support the above pictures, optical responses of the sawtooth nanoslit array are simulated by using commercial finite-difference time-domain algorithm (FDTD Solution, Lumerical) for *x* and *y* polarization excitations along both forward and backward directions^46^.

### Simulations of multiple optical paths

**Fig. 2:**
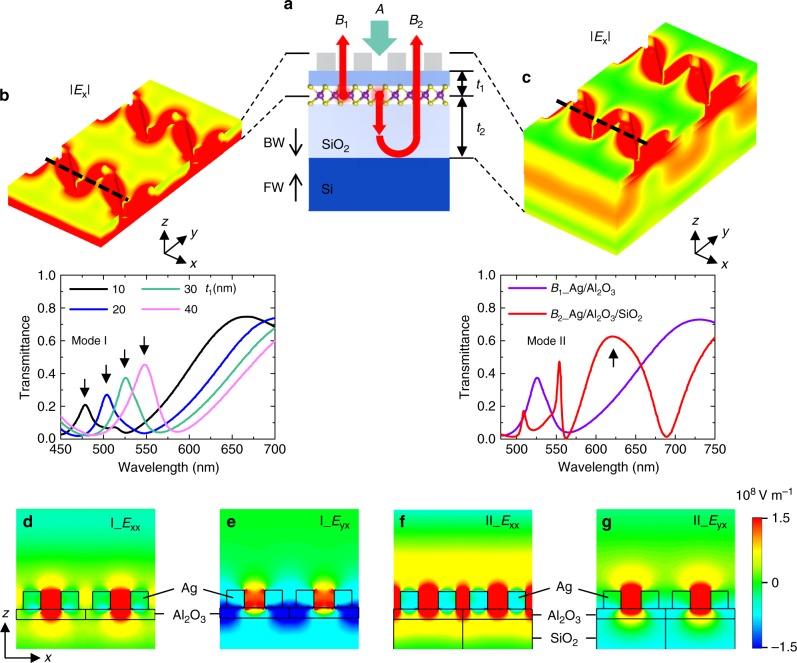
Two optical modes dominating excitation and emission paths of the WS_2_-Ag hybrid nanostructure. **a** The excitation path of 532 nm pump laser from nanoslits to WS_2_ in the backward (BW) direction is denoted as *A*, the emission path from WS_2_ to nanoslits in the forward (FW) direction as *B*_1_ and the emission path from WS_2_ to Si and bounced back to the nanoslits as *B*_2_. *t*_1_ and *t*_2_ indicate the thickness of Al_2_O_3_ and SiO_2_, respectively. **b** The BW transmittance spectra are simulated under the TE polarized light incidence for the excitation path *A*, i.e. silver nanoslits (*b* = 200 nm, *θ* = 45°) on Al_2_O_3_ with increasing *t*_1_ from 10 to 40 nm, in which the resonance mode is denoted as Mode I whose electric field distribution is presented in 3D. **c** The FW transmittance spectra of the emission paths *B*_1_ and *B*_2_, i.e. silver nanoslits and Al_2_O_3_ (*t*_1_ = 30 nm) without (violet curve) and with (red curve) SiO_2_, respectively, are calculated for the TE polarized light incidence, the latter of which exhibits a resonance mode II whose electric field distribution is also depicted in 3D. **d**–**g** Electric fields *E*_xx_ and *E*_yx_ for resonances I and II in the *xz* plane cutting along the dashed lines denoted in the 3D view (**b**, **c**).

As shown in Fig. 2a, multiple optical paths are depicted in the cross-sectional view of the device to illustrate the laser excitation and the PL emission (The color-coding is the same as that in Fig. 1a). For the excitation path *A*, to arrive at the surface of the WS_2_ monolayer, the incident 532 nm laser (TE polarized) needs to transmit through sawtooth nanoslits and the Al_2_O_3_ layer along the backward (BW) direction as indicated by the green arrow. The PL emission occurs around 630 nm which corresponds to the direct band gap transition of neutral excitons of WS_2_ monolayers (~2.0 eV) and mainly follows two optical paths as marked by *B*_1_ and *B*_2_ (red arrows). Path *B*_1_ starts from the top surface of the WS_2_ monolayer and then transmits through the Al_2_O_3_/sawtooth nanoslits along the forward (FW) direction. For path *B*_2_, PL is emitted from the bottom surface of the WS_2_ monolayer, going through the SiO_2_ layer to the top surface of the silicon substrate and then is bounced back to the sawtooth nanoslits along the forward direction, and finally penetrates the sawtooth nanoslits.

The excitation of WS_2_ flakes is realized by generating a resonant transmission mode around the wavelength of the pump laser, which is represented by the transmission peak of silver nanoslits on Al_2_O_3_ as indicated by the black arrows in Fig. 2b. The peak position shifts from 475 to 550 nm when the thickness of Al_2_O_3_ increases from 10 to 40 nm. With this continuous shift, one can always find a proper thickness allowing for the coupling of the pump laser with the WS_2_ monolayer for an efficient excitation. Here, a 30 nm thick Al_2_O_3_ was chosen in the following simulation and experiment whose transmission peak is located at ~530 nm (mode I denoted by the green curve in Fig. 2b) coinciding well with the pump wavelength. The emission of the WS_2_ monolayer is determined by the resonant transmission mode at the emission wavelength denoted as mode II in the red curve in Fig. 2c. This mode rises upon a layer of SiO_2_ is integrated under the Al_2_O_3_, which fits well with the emission path *B*_2_. As for path *B*_1_, a resonance mode is absent at the emission wavelength as depicted by the violet curve in Fig. 2c. Consequently, the emission process is expected to be dominated by path *B*_2_.

The 3D view of the electric field |*E*_x_| for the two modes is shown in Fig. 2b, c, indicating that local fields are primarily concentrated in the Al_2_O_3_ and nanoslits, respectively. To reveal the detailed process of the resonant transmission through the nanoslits, the distributions of electric fields for both components *E*_xx_ and *E*_yx_ in the *xz* plane, i.e. cutting in the middle of the unit cell, are shown in Fig. 2d–g. For mode I, *E*_xx_ is strongly localized in the slanting holes (Fig. 2d) while *E*_yx_ is oscillating in the Al_2_O_3_ layer beneath the vertical holes as shown by the dark blue regions in Fig. 2e, which is indicative of sufficient energy transfer from pump laser to the WS_2_ monolayer. For mode II, strong oscillations exists in both slanting and vertical holes for *E*_xx_ (Fig. 2f) while a relatively weak resonance locates solely in the slanting holes for *E*_yx_ (Fig. 2g), both of which promote the resonant coupling between the plasmons and the excitons. Importantly, for modes I and II, the far field scattering is dominated by *E*_xx_ rather than *E*_yx_, despite that both components can be excited in the near field (Supplementary Fig. 2). The reason lies on that *E*_yx_, which stems from the polarization conversion of the *x* polarized incidence, is mainly absorbed rather than scattered by the nanostructure.

**Fig. 3:**
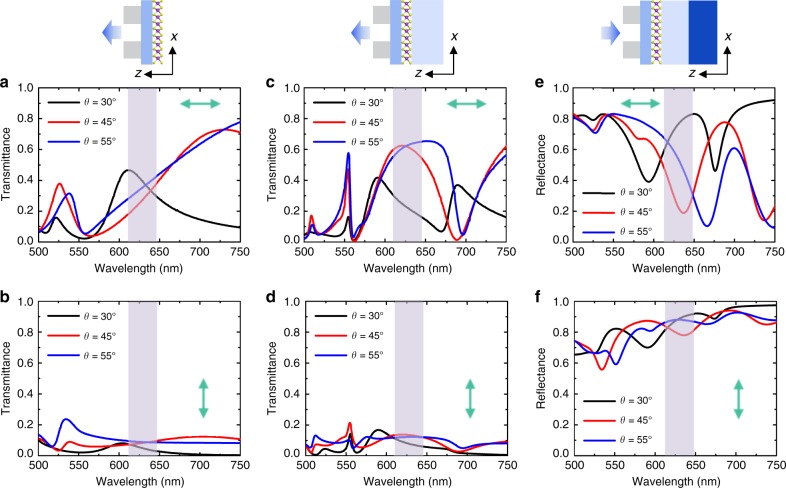
Structural dependence of polarization resolved spectra by simulations. **a**, **b** Forward transmittance spectra of the nanoslit array on Al_2_O_3_ under the TE (top row) and TM (bottom row) polarized light incidence. Structural parameters for three types of sawtooth nanoslit arrays: Black curve *θ* = 30°, *b* = 118 nm; red curve *θ* = 45°, *b* = 200 nm; blue curve *θ* = 55°, *b* = 260 nm. **c**, **d** Forward transmittance spectra of the nanoslit array on Al_2_O_3_/SiO_2_. **e**, **f** Backward reflectance spectra of the nanoslit array on Al_2_O_3_/SiO_2_/Si.

To evaluate the polarization responses of light propagating through the emission paths *B*_1_ and *B*_2_, polarization resolved optical spectra of the silver nanoslit array on different substrates are calculated as shown in Fig. 3. In addition, to modulate the resonant transmission properties, the shape of sawtooth nanoslits are engineered by tuning the variable *θ*, i.e. the angle of the middle slit relative to the *x* axis.

For emission path *B*_1_, the transmittance of silver nanoslits on Al_2_O_3_ is ~0.3 and 0.1 around 630 nm for TE (Fig. 3a) and TM (Fig. 3b) polarizations, respectively, which satisfies the requirement of anisotropic transmission. The transmittance is comparable for three types of nanoslit arrays implying that the polarization of light along the emission path *B*_1_ is less influenced by *θ*. For emission path *B*_2_, the transmittance spectra of sawtooth nanoslits on Al_2_O_3_/SiO_2_ are shown in Fig. 3c, d. The resonant transmission mode is denoted by a broad and high transmission peak (Fig. 3c) locating between two transmission minima, whose full width at half maximum (FWHM) is strongly dependent on the slanting angle, i.e. the larger *θ* leads to wider FWHM (Supplementary Fig. 3). The minima at 560 and 690 nm probably arise from SPP Bloch waves, which are determined by the high reflectance of SiO_2_/Al_2_O_3_ and Al_2_O_3_/Ag interfaces, respectively^47^. Our simulations show that the spectral positions of the two minima are blue/red shifted accordingly with decreasing/increasing the lattice spacing in the *y* direction, enabling a high tunability of the resonant transmission mode (Supplementary Fig. 4). The wide transmission peak is, however, advantageous to fit for the wide PL spectra of TMDC monolayers that are usually broadened by the disordered potential of chalcogenide vacancies and other impurities^48^. The peak position for the TE polarization is changed from 580, 630 to 670 nm when *θ* is increased from 30°, 45° to 55°, while the transmittance of the TM polarization remains low and flat, as shown in Fig. 3d. This is of utmost importance for the polarization modulation of the PL emission through the geometrical engineering of the plasmonic nanostructure.

To simulate the experimental configuration, backward reflectance spectra of three types of sawtooth nanoslits on Al_2_O_3_/SiO_2_/Si substrates are calculated as shown in Fig. 3e, f. Despite the shift of reflectance dips coincides well with that of the resonant transmission peaks in Fig. 3c, the dips become shaper when the silicon substrate is included, suggesting the enhancement of surface plasmon resonances.

In summary, different optical paths are explicitly analyzed: (1) optical mode around the pump wavelength can be excited in the nanoslits-Al_2_O_3_ nanostructure to fit the excitation path, whose spectral position is determined by the thickness of the Al_2_O_3_ layer. (2) When the SiO_2_ is assembled under the Al_2_O_3_, the optical mode is moved to the emission wavelength of WS_2_ excitons and behaves as a broad and high transmission peak polarized in the TE direction. The spectral position and FWHM of the transmission peak change dramatically with the slanting angle *θ*, while the transmittance keeps low and flat for the TM polarized incidence, resulting in a big transmittance difference between *x* and *y* polarizations at the emission wavelength. The polarized resonant transmission properties of the sawtooth nanoslit array allow for manipulating the polarization of the PL emission through a well designed plasmon-exciton hybrid system.

### Polarization modulation of the excitonic emission

**Fig. 4:**
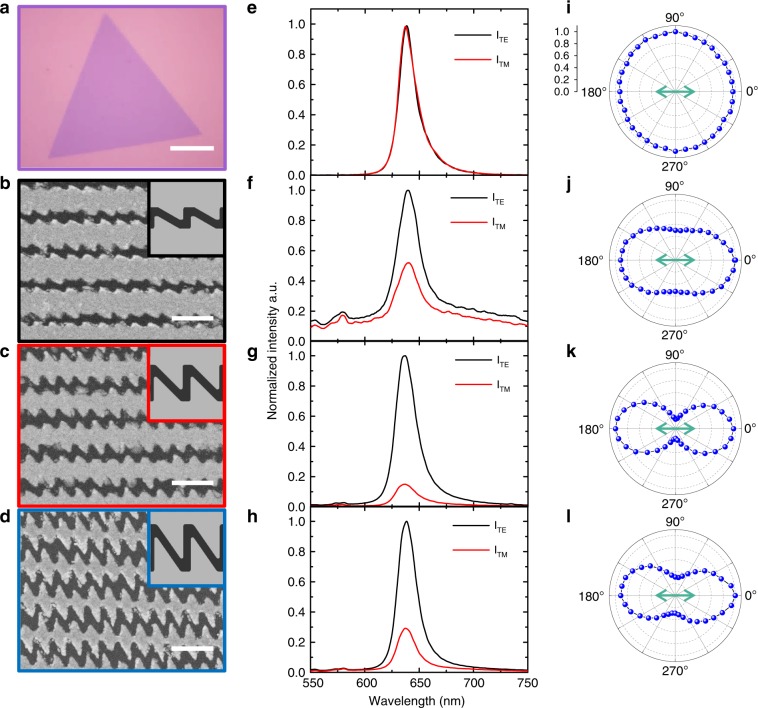
Polarization resolved PL characterization. **a** Optical image of a bare WS_2_ monolayer (scale bar is 10 μm). **b**–**d** SEM images of silver sawtooth nanoslits with *θ* = 30° (**b**), 45° (**c**) and 55° (**d**), respectively. Scale bar is 500 nm. The inserts at the top right corner show two unit cells of the corresponding array. **e**–**h** Normalized PL spectra of bare WS_2_ monolayer and three hybrid nanostructures with a peak at ~636 nm for the detection angle along TE (black curve) and TM (red curve) polarization directions respectively. **i**–**l** Normalized PL peak intensity, as a function of the detection angle for a given incident laser polarization (marked by the green arrow) for WS_2_ monolayer (**i**) and three hybrid nanostructures (**j**–**l**), respectively. 0 corresponds to the center and 1.0 to the outermost dashed line.

To verify the polarization responses of the excitonic emission, a WS_2_-Ag hybrid system is fabricated (see the Methods section). The optical image of a CVD grown WS_2_ monolayer is shown in Fig. 4a exhibiting a regular triangle shape with a uniform surface. The SEM images of three types of sawtooth nanoslits are shown in Fig. 4b–d in which the slanting angle of the middle slit is 30°, 45°, and 55°, respectively. The hybrid nanostructure was measured by the polarization resolved PL spectroscopy (see the Methods section), which was excited by a 532 nm laser along the backward direction with the incident polarization fixed in the TE (0°) direction. The emitted photons passing through the sawtooth nanoslit array were then collected by the spectrometer.

As a reference, PL spectra of a bare WS_2_ monolayer are measured. The emission in TE and TM directions are denoted by the black and red curves in Fig. 4e, respectively, where no difference could be found. The intensity is further characterized as a function of the collection angle of the analyzer. As expected, no anisotropy is observed as shown in Fig. 4i, indicating that the PL of the bare WS_2_ monolayer is indeed randomly polarized at room temperature. In contrast, with silver sawtooth nanoslits on top, the difference of the PL intensity between TE and TM is significantly enhanced as shown in Fig. 4f–h, where each graph corresponds to a specified angle of the slanted nanoslit. The angular dependences of the emission intensity are plotted in Fig. 4j–l, respectively. One would find that the difference between TE and TM is relatively small for the sawtooth nanoslit array with *θ* = 30° (Fig. 4f, j), plausibly because the resonant transmission peak at 580 nm (black curve in Fig. 3c) is far away from the emission wavelength at ~630 nm. So when the resonant transmission peak of the sawtooth nanoslit array (*θ* = 45°) is shifted to 630 nm (red curve in Fig. 3c), the striking difference is observed as shown in Fig. 4g, k. Further increase of the slanting angle, the difference starts to degrade (Fig. 4h, l). As such, by integrating silver sawtooth nanoslits above the WS_2_ monolayer, a linear polarization of the PL emission appears, and can be further optimized by tuning the geometrical parameter, and hence the resonant transmission modes of the sawtooth nanoslit array.

**Fig. 5:**
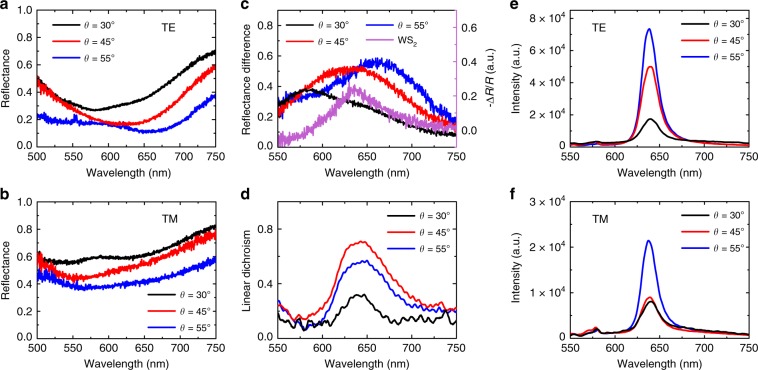
Plasmon and exciton coupling in three types of WS_2_-Ag hybrid nanostructures. Reflectance spectra are measured for the TE polarized light incidence in (**a**) and TM polarized in (**b**) for varying slanting angles. **c** Reflectance difference between TE and TM polarizations. The differential reflectivity of the WS_2_ monolayer is depicted by the purple curve. **d** Measured linear dichroism of the PL emission. **e**, **f** Intensity comparison among the three hybrid nanostructures for TE and TM polarized PL emissions, respectively.

To gain deeper understanding of the modulated polarization responses of the excitonic emission, polarization resolved reflectance spectra of three hybrid nanostructures were measured with a white light illumination. As shown in Fig. 5a, pronounced reflectance dips are observed in the spectra of the TE polarization, suggesting the excitation of the strong resonant transmission modes. In contrast, reflectance spectra along the TM polarization are higher in magnitude and more flat across the measured spectrum (Fig. 5b). Consequently, the PL emission in the TE polarization direction can be enhanced while that of the TM polarization cannot, resulting in the polarized resonant emission. We note that the measured reflectance dips (Fig. 5a) are much broader than the theoretical simulation (Fig. 3e), which can be accounted by the size variations of sawtooth nanoslits owing to the imperfect nanofabrication. In addition, the reflectance dip shifts from 580, 630, to 670 nm when *θ* is changed from 30°, 45°, to 55° as denoted by the black, red and blue curves in Fig. 5a, respectively. The shift of reflection dips is consistent with the numerical simulations (Fig. 3e), manifesting the desirable structural engineering of the metasurface. The spectral shift can be easier captured, as shown in Fig. 5c, by the reflectance difference defined as (*R*_TM_ − *R*_TE_)/(*R*_TM_ + *R*_TE_), where the magnitude, i.e. the polarization anisotropy, rises from 0.4, 0.5 to 0.6 as the slanting angle increases.

To highlight the effect of anisotropic resonances on the excitonic emission, we also plot the differential reflectivity that characterizes the absorption feature of the WS_2_ monolayer, as shown by the purple curve in Fig. 5c. The peak at 630 nm matches the direct band gap transition of the WS_2_ excitons, coinciding well with the peak of the reflectance difference for the sawtooth nanoslit array with *θ* = 45° but deviating largely from that of *θ* = 30° and 55°. Consequently, one would obtain different coupling strength between the plasmon and the exciton, leading to different linear dichroism (LD) of the PL emission $\left( {{\mathrm{LD}} = (I_{{\mathrm{TE}}} - I_{{\mathrm{TM}}})/(I_{{\mathrm{TE}}} + I_{{\mathrm{TM}}})} \right)$ as shown in Fig. 5d. The highest linear dichroism is generated by the sawtooth nanoslits with *θ* = 45° rather than 55°, despite the latter has the maximum polarization anisotropy in the plasmon mode. This is because the spectral overlap is much more crucial for the resonant coupling, through which the polarization of plasmons can be thoroughly inherited by the excitons.

To quantify the coupling with plasmonic modes, we investigate the evolution of PL intensity of three hybrid nanostructures for TE and TM polarizations as a function of the slanting angle. For TE polarization, the hybrid nanostructure with *θ* = 30° has the lowest PL as denoted by the black curve in Fig. 5e. This is due to the fact that the nanoslit array with 30° slanting angle has a high reflectance at the pump wavelength (black curve in Fig. 3a) and a low transmittance at the emission wavelength (black curve in Fig. 3c), leading to the less efficient excitation and emission. The intensity increases dramatically (2.8 times) when the slanting angle *θ* is changed to 45° (red curve in Fig. 5e), which is attributed to the better coupling of WS_2_ excitons with the resonant transmission modes of sawtooth nanoslits at both laser excitation (red curve in Fig. 3a) and PL emission (red curve in Fig. 3c) wavelengths. The PL intensity exhibits the largest enhancement (4.2 times) when *θ* is increased to 55° as shown by the blue curve in Fig. 5e, even though the simulated transmittance is not increased as much (blue curve in Fig. 3c). The discrepancy can be ascribed to the increase of the slit width in the experiment, which are narrower and fixed for three hybrid structures in the simulation, as wider slits allow more energy passing through therefore leading to stronger emission.

For TM polarization, the PL intensity is much lower than that of the TE polarization. Specifically, the lowest PL intensity is from the nanostructure of *θ* = 30° due to the large deviation of the transmission mode from the emission wavelength and the lowest transmittance among the three nanostructures as shown in Fig. 3b, d. The PL intensity remains extremely low when *θ* is increased to 45°, which is six-fold lower than that of the TE polarization (red curve in Fig. 5f). It suggests the emission in the TM polarization direction can be effectively suppressed by choosing a proper geometrical parameter of the sawtooth nanoslit array, while the emission in the TE polarization direction keeps being high, thereby leading to the largest linear dichroism as shown by the red curve in Fig. 5c. In case of *θ*  = 55°, despite the PL intensity in the TM polarization direction is 2.4 times that of *θ*  = 45°, the PL in the TE direction is merely 1.5 times (blue curve in Fig. 5e), hence the linear dichroism is actually decreased. In short, to increase the linearity of the PL emission, one needs to enhance the PL emission in one polarization (TE here), and minimize it in the perpendicular direction at the same time, both of which require the coherent coupling between excitons and anisotropic resonant transmission modes.

### Linear polarization from the valley coherence

**Fig. 6:**
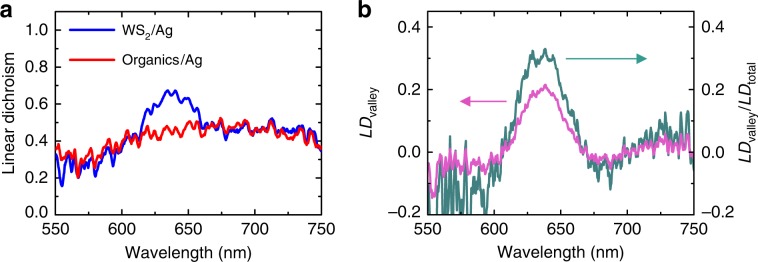
Resolving the polarization induced by the valley coherence from the total polarization. **a** Comparison of the linear dichroism between two kinds of hybrid nanostructures. The linear dichroism of the WS_2_-Ag hybrid is always higher than that of the organics-Ag hybrid at the emission wavelength of the valley excitons (~636 nm). The organics is primarily the fluorescent moieties of PMMA residue. **b** The linear dichroism induced by the valley coherence (pink curve) and its percentage in the total polarization (green curve). Note that ${\mathrm{LD}}_{{\mathrm{total}}} = {\mathrm{LD}}_{{\mathrm{WS}}_2/{\mathrm{Ag}}}$.

Besides the anisotropic resonant transmission modes, the other contributor to the large linear polarization is the coherent superposition of the excitonic emission from *K*/*K*′ valleys, which is enhanced by coupling of the valley excitons with the ultrafast plasmonic nanocavity. To extract the contribution from the valley coherence, we compare the PL spectra of the sawtooth nanoslit array with and without integration of the WS_2_ monolayer. In the bare array, the fluorescent moieties of the embedded PMMA residue show broad PL, whose polarization reflects purely the anisotropic transmittance of the metasurface since valley is absent in this organics. As shown in Fig. 6a, the linear dichroism is compared for both WS_2_-Ag and organics-Ag hybrid nanostructures by measuring the PL spectra along TE and TM polarization directions (Supplementary Fig. 5 and Supplementary Note 1). At the excitonic emission of the WS_2_ monolayer, the linear dichroism of the WS_2_-Ag hybrid is clearly higher than that of the organics-Ag hybrid, while they almost coincide with each other elsewhere. It is easy to understand the coincidence across most of the measured spectra, as they both originate from the anisotropic transmittance of the nanostructure. However, the difference—the peak around 636 nm—must be related to the intrinsic valley properties of WS_2_ itself, which can be attributed to the plasmon enhanced valley coherence. The polarization from the valley coherence can be evaluated quantitatively by ${\mathrm{LD}}_{{\mathrm{valley}}} = {\mathrm{LD}}_{{\mathrm{WS}}_2/{\mathrm{Ag}}}-{\mathrm{LD}}_{{\mathrm{Organics}}/{\mathrm{Ag}}}$, which is ~0.2 (pink curve in Fig. 6b) and contributes about 30% of the total linear dichroism (green curve).

## Discussion

In a recent theoretical work, two distinct routes are proposed to overcome the quantum decoherence^43^. One is to increase the coherence at low temperatures or to protect the coherence through optical microcavities^41,42,49,50^; the other is the plasmonic speed-up approach to enhance the light-matter coupling by using highly localized electromagnetic modes of the metallic nanostructures. Here, in our hybrid system, it is attributed to the latter mechanism. The resonant mode of the sawtooth nanoslit array is plausible to accelerate the electron-hole recombination rate^51,52^, which, whenever faster than that of quantum decoherence events, would guarantee the valley coherence during excitons’ lifetime. To confirm the generality of the plasmonic origin, we carried out a similar measurement but in an isotropic metasurface, i.e. a WS_2_-silver nanodisk array hybrid nanostructure. It is found the plasmon enhanced valley coherence remains robust, resulting in LD_valley_ ~ 0.1–0.14 (Supplementary Fig. 6 and Supplementary Note 1). The higher magnitude of LD_valley_ up to 0.27 in the sawtooth nanoslit array (Supplementary Fig. 5l) is probably due to the stronger coupling of valley excitons with the resonant transmission mode of the sawtooth nanoslit array, as manifested by the sharper reflectance dip in the red curve of Fig. 5a. Compared with all values reported previously, the present valley coherence at room temperature can even rival the low temperature performance (Supplementary Table 1).

It is worth noting the resulted linear dichroism from the valley coherence is far from the criteria in practice, consequently, the anisotropic resonant transmission mode is required to further boost the polarization significantly. A large linear dichroism requires that the emission intensity in the TE direction is high enough and that in the TM direction is as low as possible. The increase of the PL intensity can be achieved by either shifting the resonant transmission peak closer to the emission wavelength or increasing the width of the sawtooth nanoslits. The former allows not only the multifold increase of the PL intensity in one polarization direction but also the suppression of the emission in the perpendicular direction. In contrast, the latter increases the PL intensity for both polarizations. As a result, it is preferred to obtain a high linearity by establishing anisotropic resonant transmission modes just in resonance with the WS_2_ excitons and then the emission intensity can be balanced by the linewidth of the nanoslits.

Now let’s return to the choice of sawtooth element in the nanoslit array. Different from the conventional line nanoslit array whose transmission peak induced by the surface plasmon resonance is quite narrow and responds primarily to the TM polarization excitation^53^, sawtooth nanoslit array allows the broadband resonant transmission of the TE polarized light. The broad bandwidth is an advantage for the easy and fast coupling of the plasmonic resonance with the valley excitons, so that the valley coherence can be efficiently boosted. Especially, the linewidth and the spectral position of the transmission peak can be controlled accurately by the geometrical parameters, e.g. the *θ* and period here. This enables not only the highly efficient PL emission but also the broadband modulation of the polarization, which is difficult to be achieved in the line nanoslit array with the similar structural parameters (Supplementary Figs. 7 and 8 and Supplementary Note 2).

In conclusion, polarization modulation of the excitonic emission is demonstrated in a hybrid nanostructure consisting of a WS_2_ monolayer and a sawtooth nanoslit array, where coupling the resonant transmission modes with both excitation and emission fields are essential. While the highly localized E-field resonance at the pump wavelength enables efficient PL excitation, the polarized resonant transmission mode around the emission wavelength allows strongly polarized PL emission through the resonant coupled transition. This is due to the ability to enhance the PL emission along the TE while suppressing it in the TM polarization direction. Furthermore, the plasmon-exciton coupling is found to enhance the valley coherence substantially, resulting in a linear dichroism up to 0.27 at room temperature. As a result, the total linearity can be optimized to ~80% in experiments. Our study provides a good opportunity for the exploration of light-matter interaction in the plasmon-exciton hybrid system based on TMDCs, paving the way to the development of 2D polaritonic devices for polarization applications in the integrated nanophotonics.

## Methods

### Materials growth

Tungsten disulfide monolayers were synthesized via chemical vapor deposition method. Tungsten triple oxide (99.995%) and sulfur (99.998%) powders were used as the source, silicon wafers with 270 nm SiO_2_ on top as substrates, and argon as carrying gas. Firstly, the quartz tube was flushed by a high flow rate of high-purity argon for 20 min to remove the air completely. Then the argon flow rate was set as 100 standard-state cubic centimeter per minute (sccm), and the furnace was rapidly heated to 850 °C for tungsten triple oxide at 30 K/min and a separate heater heats the sulfur source to 190°. This growth condition lasts for 10 min, followed by natural cooling down to room temperature.

### Device fabrication

The Al_2_O_3_ layer (30 nm in thickness) was firstly deposited on the WS_2_ monolayer by e-beam evaporation with a rate of 1 Å s^−1^. Then the sawtooth slit arrays were patterned on Al_2_O_3_ by the state of art e-beam lithography^54^ using negative e-beam resist Ma2403 (Micro resist technology). After development with the developer D525, the sample was flushed with DI water for ~5 min and dried by nitrogen gas. The metallic sawtooth nanoslit array was formed by e-beam evaporation of 50 nm silver followed by liftoff process using acetone.

### Polarization resolved PL/white light spectral measurement

The excitation laser was 532 nm polarized in the TE direction, whose power was measured to be 160 μW under the ×50 objective lens. The PL emission was collected by the spectrometer (Andor SR-500) and CCD camera (iDus 420). The polarization of the PL emission is analyzed by placing a linear polarizer before the 532 nm edge filter and rotating it from zero to 360° with 0° (TE)/90° (TM) defined as the angle parallel/perpendicular to the long axis of the nanoslits array. For white light spectral measurement, the polarization of the incident white light was controlled by placing a polarizer between the white light source and the sample, and then the reflected light was collected by the same spectrometer.

## Supplementary information

Supplementary Information

## Data Availability

The data that support the findings of this study are available from the corresponding author upon request. The source data underlying Figs. 2b, c, 3, 4e–l, 5, 6 and Supplementary Figs. 1, 3, 4, 5, 6b–k, 7b–e, 8 are provided as a Source Data file.

## Source data

Source Data
