# Supplementary material for: Polarized resonant emission of monolayer WS2 coupled with plasmonic sawtooth nanoslit array
Source: Nat Commun. 2020 Feb 5;11:713. doi: 10.1038/s41467-020-14597-2 (PMC7002612; doi:10.1038/s41467-020-14597-2)
Supplement: Supplementary file 1 — Supplementary Information [file 41467_2020_14597_MOESM1_ESM.pdf]

# Supplementary Information

## Polarized resonant emission of monolayer $WS_2$ coupled with plasmonic sawtooth nanoslit array

Han et al.

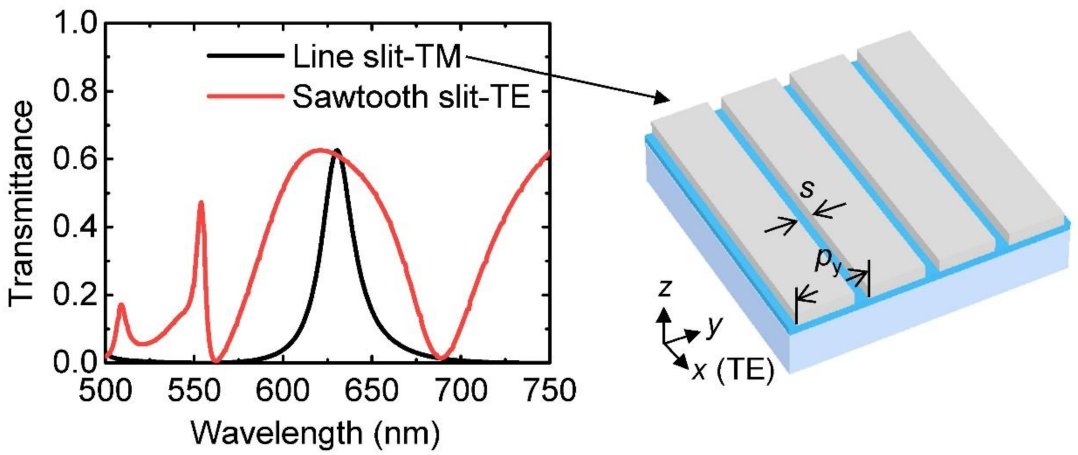


**Supplementary Figure 1.** Comparison of the resonant transmission modes around the emission wavelength between the sawtooth and the line nanoslit array with the same period, slit width and thickness, on the $Al_2O_3/SiO_2$ substrate. The full width at half maximum (FWHM) is 78 and 21 nm, respectively. Schematic view of the line nanoslit array is shown on the right hand side with $p_y$ = 400 nm, $s$ = 40 nm.

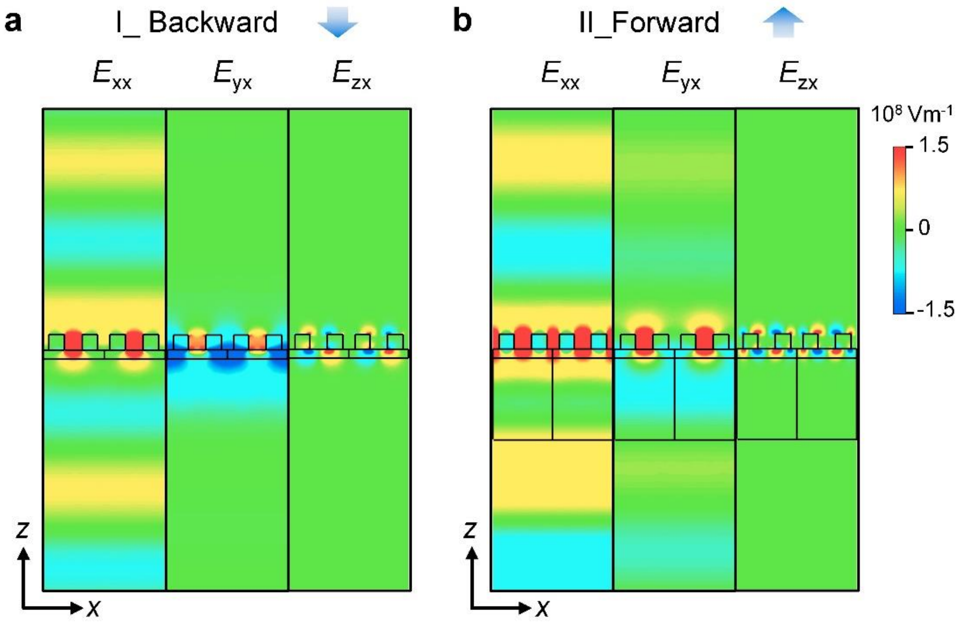


**Supplementary Figure 2.** Distributions of the electric fields $E_{xx}$, $E_{yx}$ and $E_{zx}$ in the far field for modes I and II in the *xz* plane. **a** For mode I, the resonance of $E_{xx}$ allows the propagation of the electric field along either forward or backward direction, while the resonance of $E_{yx}$ is tightly bounded by the nanostructure without any scattering. **b** For mode II, the forward scattering is very strong for $E_{xx}$, while that of $E_{yx}$ is extremely weak. The behaviors for both modes imply that $E_{xx}$ dominates the far field scattering, which hence allows the polarized resonant transmission of the PL emission.

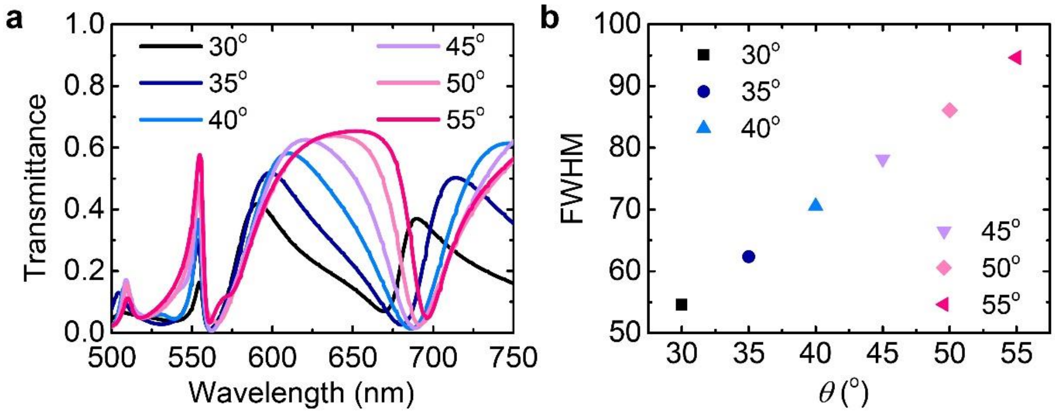


**Supplementary Figure 3.** The resonant transmission mode II as a function of the slanting angle of the middle slit. **a** The resonant transmission spectra of the sawtooth nanoslit array on the $Al_2O_3/SiO_2$ substrate for slanting angles $\theta$ ranging from 30$^o$ to 55$^o$. **b** Full width at half maximum (FWHM) of the mode II as a function of the slanting angle $\theta$ of the middle slit.

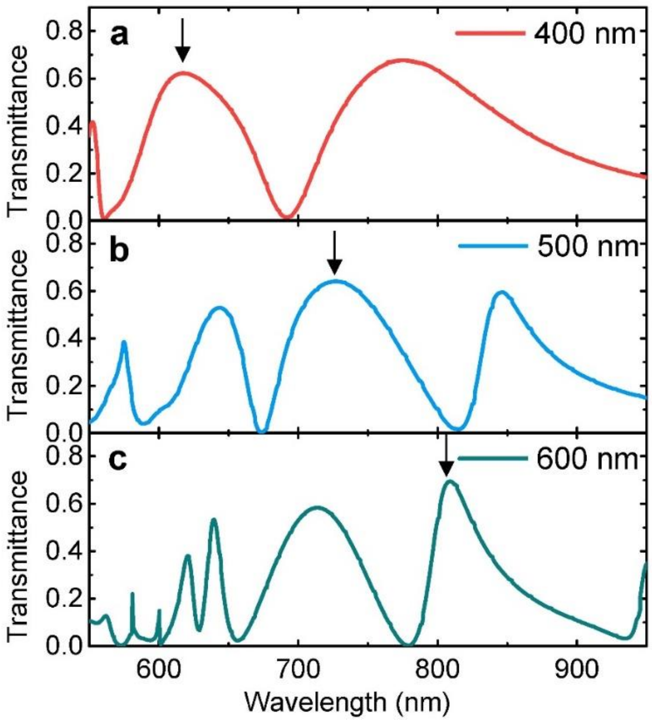


**Supplementary Figure 4.** The resonant transmission mode II as a function of the lattice constant along the *y* direction $p_y$. **a-c** The transmission spectrum of the sawtooth nanoslit array on the $Al_2O_3/SiO_2$ substrate for $p_y$ varying from 400 to 600 nm. The resonant transmission mode red shifts from 630, 720 to 810 nm accordingly (denoted by black arrows).

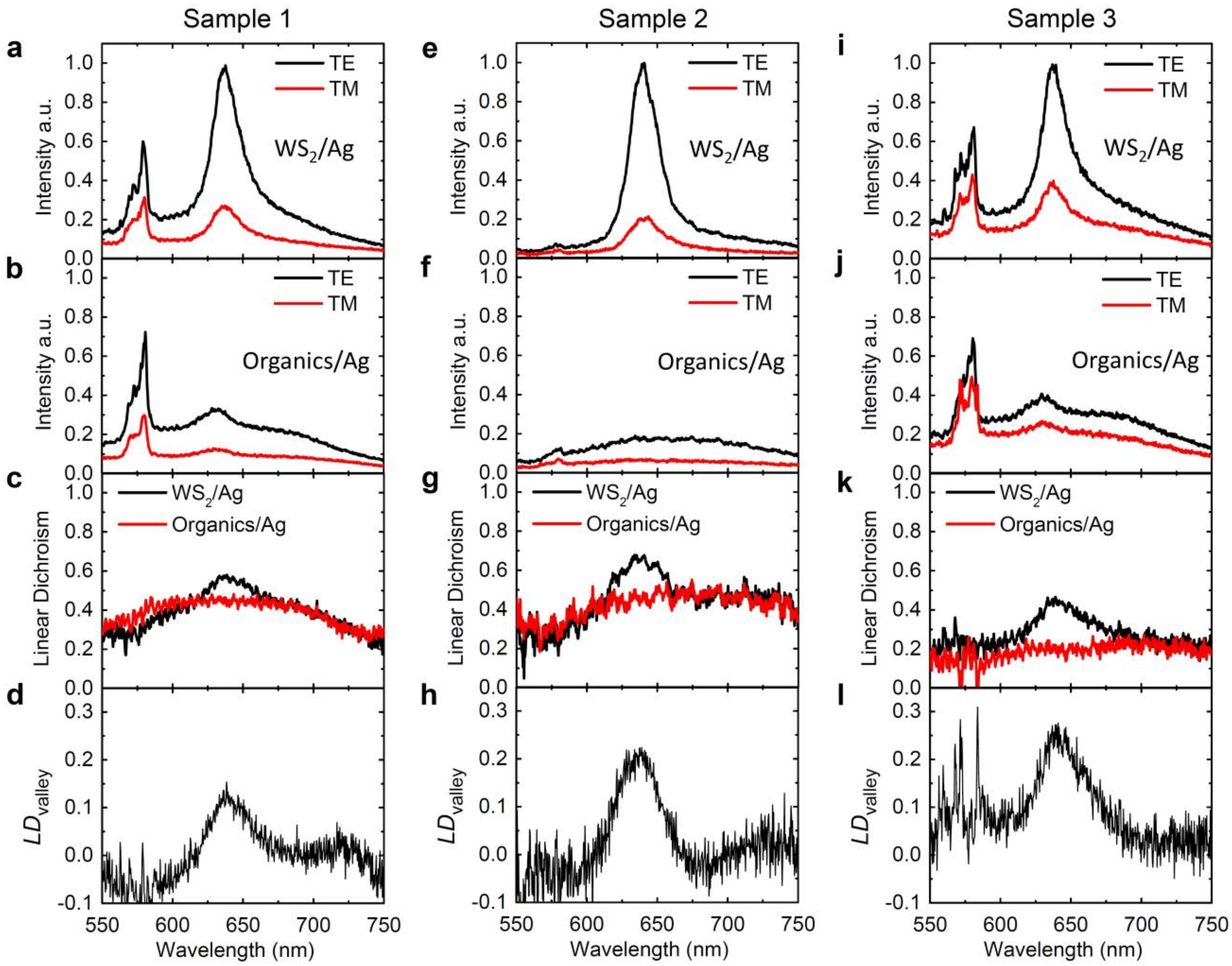


**Supplementary Figure 5.** Resolving the polarization induced by the valley coherence. **a, b** Normalized PL spectra along TE and TM polarization directions for $WS_2$-Ag and organics-Ag hybrids, respectively. The organics refers to the fluorescent moieties of PMMA residues. **c** Comparison of the linear dichroism (LD) between the two kinds of hybrids. **d** The linear dichroism induced by the valley coherence, defined as $LD_{\mathrm{valley}} = LD_{\mathrm{WS_2/Ag}} - LD_{\mathrm{Organics/Ag}}$. The second (e-h) and third (i-l) columns show the results for samples 2 and 3. The linear dichroism of the $WS_2$-Ag hybrid is always higher than that of the organics-Ag hybrid at the emission wavelength of the valley excitons. The excessive linear dichroism above the background, i.e. 0.14 (d), 0.20 (h) and 0.27 (l), is indicative of the valley coherence.

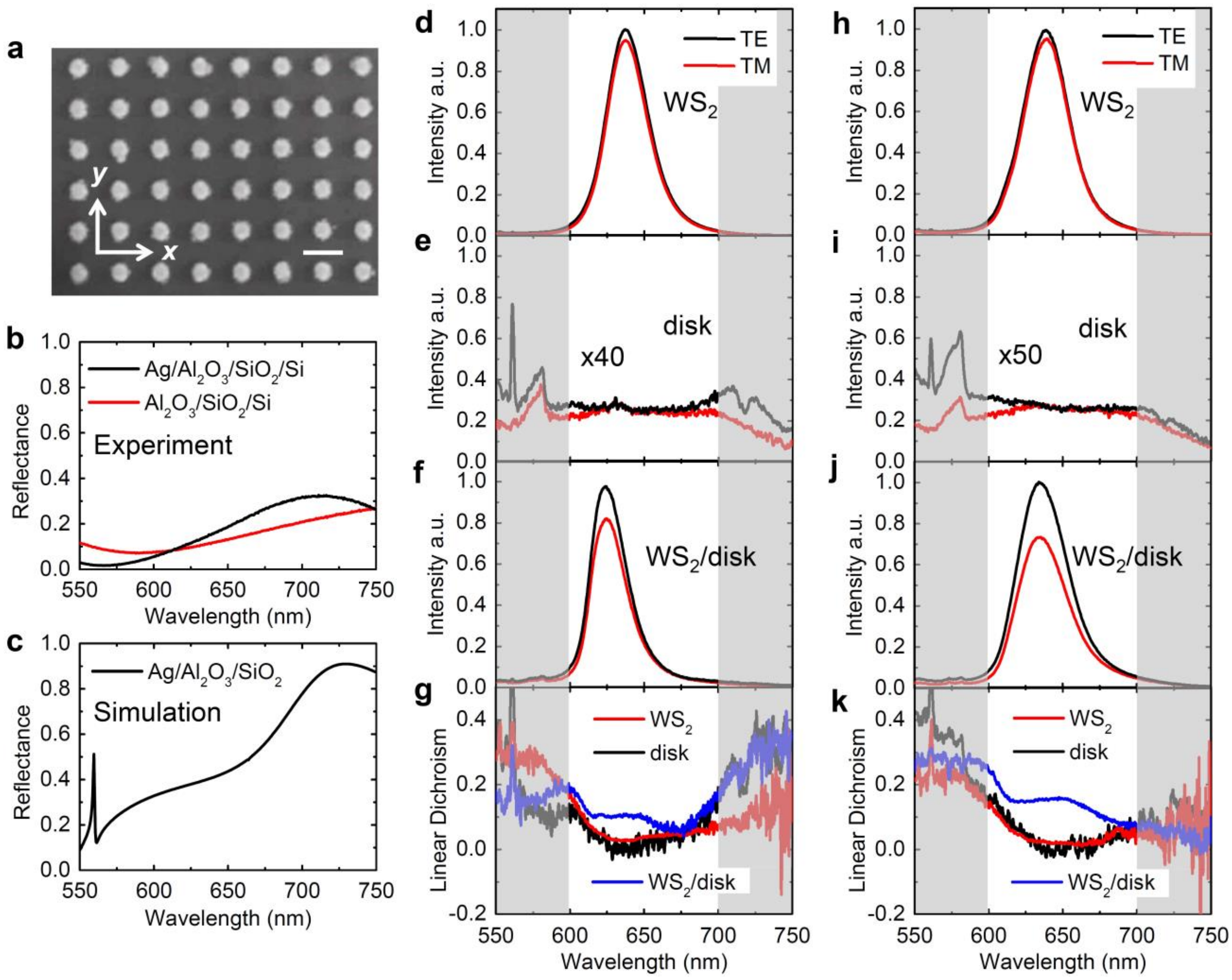


**Supplementary Figure 6.** Polarization characterization of the $WS_2$-nanodisk array hybrid nanostructure. **a** SEM image of the silver nanodisk array whose period is 400 nm and the diameter/thickness of a nanodisk is 150/50 nm, respectively. Scale bar is 400 nm. **b** Measured reflectance of the $Al_2O_3/SiO_2/Si$ substrate with (black curve) and without (red curve) disk array. The incident polarization of the white light illumination is in parallel with the *y*-axis of the disk array. **c** Simulated reflectance of the silver disk array on the $Al_2O_3/SiO_2$ substrate. **d-f** Polarization resolved PL spectra of the bare $WS_2$ (d), nanodisk array (e) and $WS_2$ integrated with the nanodisk array (f) respectively, for which the *y*-axis of the disk array is in parallel with the polarization of the laser excitation. **g** The corresponding linear dichroism. The higher *LD* around the valley excitonic emission wavelength (blue curve) results from the valley coherence. **h-k** Polarization

resolved PL spectra (h-j) and linear dichroism (k) when the *x*-axis of the disk array is in parallel with the polarization of the laser excitation. The spectral signals outside the valley excitonic emission region are extremely small, which are mainly from the organic residues. The large linear dichroism in these regions may be contributed by the strong dipolar resonances around 570 and 770 nm (black curve in b), which is beyond the scope of this paper and shaded by the grey color.

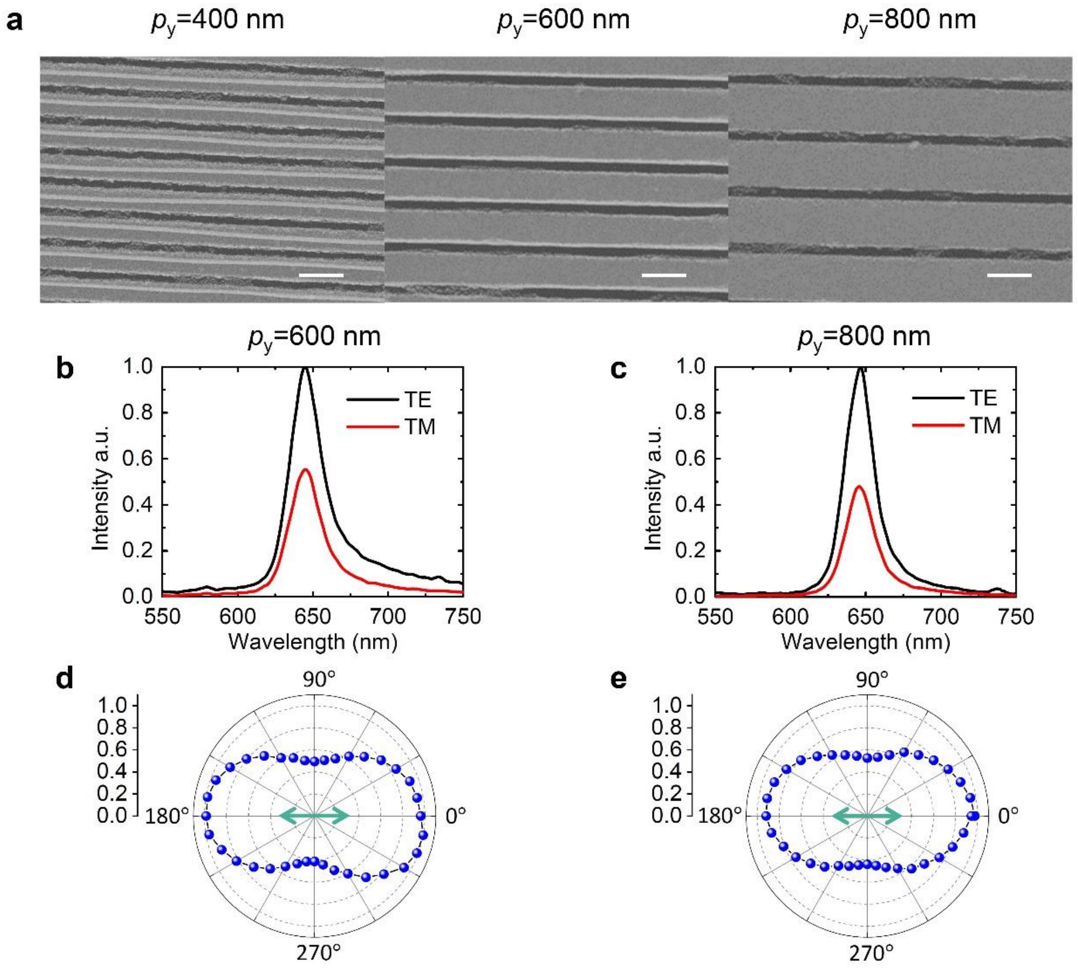


**Supplementary Figure 7.** Polarization resolved PL characterization for the $WS_2$-line nanoslit hybrid nanostructures. **a** SEM images of the line nanoslit array with period 400, 600 and 800 nm respectively. Scale bar is 600 nm. **b, c** Normalized PL spectra of the $WS_2$-line nanoslits hybrid nanostructure with periods 600 and 800 nm for the detection angle along TE and TM polarization directions, respectively. **d, e** Normalized PL peak intensity, as a function of the detection angle for a given incident laser polarization (marked by the green arrow) for the hybrid nanostructure with period 600 and 800 nm, respectively.

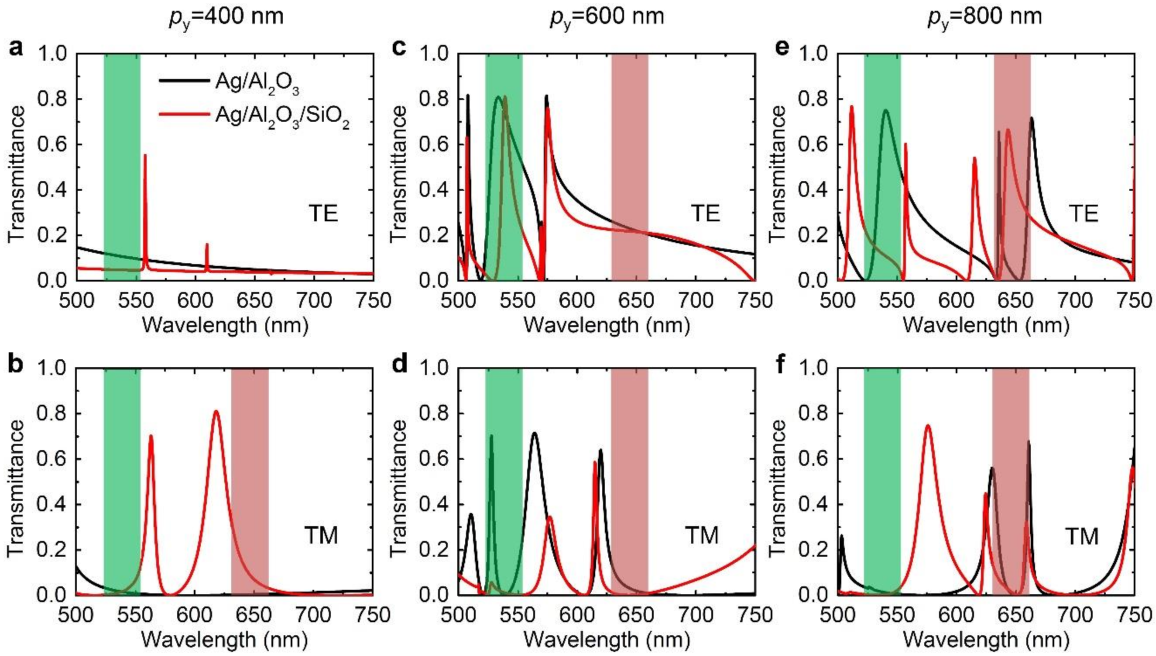


**Supplementary Figure 8.** Simulated transmittance for the line nanoslit array with period 400 (left column), 600 (middle column) and 800 nm (right column) on the $Al_2O_3$ (black curve) and $Al_2O_3/SiO_2$ (red curve) substrates. The top/bottom row is for the TE/TM polarization incidence, respectively. Green/red regions indicate the spectral positions of laser excitation and the PL emission.

**Supplementary Table 1: Representative performance of the valley polarization and the valley coherence for TMDC monolayer devices.**

| Device structure | $T$ (K) | DOCP | DOLP | $\lambda$* (nm) | Monolayer preparation | Ref. |
|---|---|---|---|---|---|---|
| $WSe_2/SiO_2/Si$ | 30 | - | 0.40 | 660 | mechanical exfoliation | 1 |
| $WSe_2/SiO_2/Si$ | 4 | - | 0.42 | 656 | mechanical exfoliation | 2 |
| $WSe_2/SiO_2/Si$ | 4 | - | 0.15 | 632 | mechanical exfoliation | 3 |
| $WS_2/SiO_2/Si$ | 4.2 | - | 0.30 | 561 | mechanical exfoliation | 4 |
| Microcavity-$MoSe_2$ | 4.2 | 0.05 | - | 638 | mechanical exfoliation | 5 |
| Microcavity-$WSe_2$ | 4.2 | - | 0.25 (LP*), 0.40 (UP*) | 638 | mechanical exfoliation | 6 |
| Microcavity-$WS_2$ | RT | 0.10 | - | 568 | mechanical exfoliation | 7 |
| Microcavity-$WS_2$ | RT | 0.27 | - | 626 | mechanical exfoliation | 8 |
| Microcavity-$MoS_2$ | RT | 0.075 (UP) 0.13 (LP) | - | 640 | CVD | 9 |
| Metasurface-$WS_2$ | RT | - | up to 0.27 | 532 | CVD | This work |

*$\lambda$: excitation wavelength, LP: Lower polariton, UP: Upper polariton.

**Supplementary Note 1: Two control systems to verify the valley contribution to the linear dichroism**

For the first control system, by fixing the metasurface to sawtooth nanoslit array, we compare the PL emission from the emitters with and without valley physics. The PL spectra of the sawtooth nanoslit array ($\theta = 45^{o}$) with and without integration of the $WS_2$ monolayer are measured as shown in Supplementary Figure 5a, b, respectively. The emission in the TE direction (black curve) is much higher than that along the TM direction (red curve). It is found that the organics, such as fluorescent moieties of PMMA residues embedded in the nanohole array can generate obvious PL signal (Supplementary Figure 5b) whose polarization reflects only the anisotropic transmission property of the metasurface since valley physics is absent in this organics. The linear dichroism calculated as $LD = (I_{TE} - I_{TM})/(I_{TE} + I_{TM})$ is shown in Supplementary Figure 5c, indicating that $LD$ of the $WS_2$-Ag hybrid is higher than that of the organics-Ag hybrid at the valley excitonic emission wavelength (~ 640 nm), while they are at the same level elsewhere. Since the polarization of the organics-Ag hybrid is only from the anisotropic nanostructure, the higher linear dichroism of the $WS_2$-Ag hybrid could be soly attributed to the valley coherence owning to the plasmon-exciton coupling. The $LD$ from the valley coherence is calculated as $LD_{\mathrm{valley}} = LD_{\mathrm{WS_2/Ag}} - LD_{\mathrm{Organics/Ag}}$, which is 0.14 for sample 1.

Two more samples were measured following the same procedure (middle and right columns in Supplementary Figure 5). We confirmed that valley coherence is universal in $WS_2$/Ag hybrid devices and similar enhancements in *LD*s were found in Supplementary Figure 5c, g and k. To be quantitative, the *LD* peaks show different enhancements of 0.14, 0.2, and 0.27 for samples 1, 2 and 3, respectively, suggesting that the contribution from valley coherence is sensitive to the device details, which is yet to be fully controlled experimentally.

For the second control system, we prepared a reference metasurface without polarization preference to show that the isotropic plasmonic nanostructure is able to preserve the valley coherence at room temperature. We change the top metasurface to nanodisk array (Supplementary Figure 6a), whose periods along both *x* and *y* directions are 400 nm and the diameter/thickness of a nanodisk is 150/50 nm. A broad reflectance dip is observed around 570 nm for the nanodisk array on the $Al_2O_3/SiO_2/Si$ substrate as shown by the black curve in Supplementary Figure 6b. It is attributed to both the broad absorption of the $Al_2O_3/SiO_2/Si$ substrate (red curve in Supplementary Figure 6b) and the low reflectance of the nanodisk array (Supplementary Figure 6c).

Without the anisotropic optical mode, the valley coherence would be the only mechanism of the linearly polarized emission. The polarization resolved PL spectra of bare $WS_2$, nanodisk array and $WS_2$ integrated with the nanodisk array were measured respectively as shown in Supplementary Figure 6d-g, in which the *y*-axis of the nanodisk array is in parallel with the polarization of the laser excitation. The *LD*s at room temperature for the three representative locations are compared in Supplementary Figure 6g. Consistently, the *LD* is almost zero around the valley excitonic emission (640 nm) for both the bare $WS_2$ (red curve) and the nanodisk array (black curves). In contrast, it increases to ~ 0.10 for $WS_2$-disk array hybrid nanostructure. Without the contribution from the bare $WS_2$ and the anisotropic plasmonic mode, this additional degree of linearity can be attributed to the coherent coupling of the $WS_2$ and the nanodisk array in which electron-hole recombination rate is accelerated by the plasmonic resonance to compete with the rate of the valley decoherence.

In Supplementary Figure 6h-k, we show a similar set of measurements of polarization resolved PL spectra excited by aligning the *x*-axis of the nanodisk array along the laser polarization. The

linear dichroism behaves similarly and the linearity from the valley coherence is ~ 0.14. The comparison above indicates that the valley coherence can survive in different plasmon-TMDC hybrid systems as long as the valley excitons couple with the plasmonic resonance.

In summary, the large linearity observed in our system arises partially from the valley coherence due to the coupling between the valley excitons and the plasmonic nanocavity rather than entirely from the anisotropic transmittance of the nanostructure. However, the polarization induced by the valley coherence is far from being sufficient in practical applications. Therefore, it is necessary to utilize the anisotropic surface plasmon mode to further enhance of the linearity of the excitonic emission.

**Supplementary Note 2: Polarization responses of the $WS_2$-line nanoslit hybrid nanostructures**

To show the polarization responses, we have made $WS_2$-line nanoslit hybrid nanostructures with different periods including 400, 600 and 800 nm. The experimental measurements and the numerical simulations are shown in Supplementary Figure 7, 8.

For $P_y$ = 400 nm, we can’t observe any PL emission experimentally. The simulation indicates that the transmittance of the array is ultralow around 532 nm for both TE (Supplementary Figure 8a) and TM (Supplementary Figure 8b) polarizations, implying that the incident laser is strongly reflected by the metasurface resulting in an inefficient excitation. Hence, the emission is not observable.

For $P_y$ = 600 nm, the PL of the hybrid nanostructure is observable, but the degree of linearity is much smaller (Supplementary Figure 7b, d) than that of the sawtooth nanoslit array (Fig. 4g in the manuscript). The simulation indicates that the metasurface allows the transmission of the incident laser through a transmittance peak around 532 nm for the PL excitation (black curve in

Supplementary Figure 8c). The emission around 640 nm is allowed, but only with moderate transmittance of the line nanoslit array (red curve in Supplementary Figure 8c). Consequently, the difference between TE and TM polarizations can't be as large as that of the sawtooth nanoslit array, thus the linearity of the PL emission is small.

For $P_y$ = 800 nm, the difference between TE and TM polarizations for the PL emission is quite similar to the sample of $P_y$ = 600 nm (Supplementary Figure 7c, e). In the simulation shown in Supplementary Figure 8e, although the optical modes support the excitation and the PL emission around 532 (black curve) and 640 nm (red curve) respectively, the transmittance at the emission wavelength is quite narrow, making it difficult to couple the excitons with the transmittance mode.

Overall, despite that we could observe a difference between TE and TM polarizations in the $WS_2$-line nanoslit array, the linearity is much lower than that from the sawtooth array. The physical origin is that the transmission resonance is either narrow or very weak, making the simultaneous coupling to the excitation and the emission paths more difficult to realize.

**References**


1. Jones, A. M. et al. Optical generation of excitonic valley coherence in monolayer $WSe_2$. *Nat. Nanotechnol.* **8**, 634–638 (2013).
2. Wang, G. et al. Double resonant raman scattering and valley coherence generation in monolayer $WSe_2$. *Phys. Rev. Lett.* **115**, 117401 (2015).
3. Wang, G. et al. Control of exciton valley coherence in transition metal dichalcogenide monolayers. *Phys. Rev. Lett.* **117**, 187401 (2016).
4. Schmidt, R. et al. Magnetic-field-induced rotation of polarized light emission from monolayer $WS_2$. *Phys. Rev. Lett.* **117**, 077402 (2016).

5. Dufferwiel, S. et al. Valley-addressable polaritons in atomically thin semiconductors, *Nat. Photonics.* **11**, 497–501 (2017).

6. Dufferwiel, S. et al. Valley coherent exciton-polaritons in a monolayer semiconductor. *Nat. Commun.* **9**, 4797 (2018).

7. Lundt, N. et al. Observation of macroscopic valley-polarized monolayer exciton-polaritons at room temperature. *Phys. Rev. B* **96**, 241403 (2017).

8. Sun, Z. et al. Optical control of room-temperature valley polaritons. *Nat. Photonics* **11**, 491−496 (2017).

9. Chen, Y. J., Cain, J. D., Stanev, T. K., Dravid, V. P. & Stern, N. P. Valley-polarized exciton–polaritons in a monolayer semiconductor. *Nat. Photonics* **11**, 431−436 (2017).
